# Neotypification of *Protoparmeliopsis garovaglii* and molecular evidence of its occurrence in Poland and South America

**DOI:** 10.3897/mycokeys.57.34501

**Published:** 2019-08-01

**Authors:** Katarzyna Szczepańska, Pamela Rodriguez-Flakus, Jacek Urbaniak, Lucyna Śliwa

**Affiliations:** 1 Department of Botany and Plant Ecology, Wroclaw University of Environmental and Life Sciences, pl. Grunwaldzki 24a, PL-50–363 Wrocław, Poland University of Environmental and Life Sciences Wrocław Poland; 2 Laboratory of Molecular Analyses, W. Szafer Institute of Botany, Polish Academy of Sciences, Lubicz 46, PL-31–512 Kraków, Poland W. Szafer Institute of Botany, Polish Academy of Sciences Kraków Poland; 3 Department of Lichenology, W. Szafer Institute of Botany, Polish Academy of Sciences, Lubicz 46, PL-31–512 Kraków, Poland Wroclaw University of Environmental and Life Sciences Wrocław Poland

**Keywords:** Geographical distribution, ITS rDNA, lichenised fungi, phylogeny, taxonomy, typification

## Abstract

*Protoparmeliopsis garovaglii* is a widely distributed placodioid lichen, which develops a distinctly rosette thallus, composed of elongated and strongly inflated to sinuous-plicate lobes. The taxon is characterised by high morphological plasticity and varied composition of secondary metabolites. However, the epithet was never typified. As such, the identity of *P. garovaglii*, in its strict sense, was unknown for a long time. Our phylogenetic ITS rDNA analyses, including newly generated sequences, show that European (Austria, Poland), North American (USA) and South American (Bolivia, Peru) specimens of *P. garovaglii* are placed in a strongly supported monophyletic clade, sister to *P. muralis*. We provide the first molecular evidence of the occurrence of *P. garovaglii* in South America (Bolivia and Peru) and the second record in Central Europe (Poland) was also provided. Furthermore, we neotypify *P. garovaglii* and it is reported here for the first time from Poland.

## Introduction

The genus, *Protoparmeliopsis* Choisy, belongs to the large family of lichenised fungi Lecanoraceae. It includes species with a placodioid or umbilicate type of thallus, growing on siliceous rocks or on soil (Zhao et al. 2016). They produce lecanorine apothecia and *Lecanora*-type asci, specifically containing hyaline, simple ascospores. Their centre of distribution is concentrated in semi-arid regions of the northern Hemisphere. Although well established at present, owing to their treatment by Zhao et al. (2016), the history of the genus taxonomy and nomenclature is very complicated.

The *Protoparmeliopsis* genus was proposed by Choisy in 1929 with *Protoparmeliopsis muralis* indicated as a type species. However, the generic concept was not followed and, consequently, the majority of the lecanoroid species with characteristic placodioid thallus morphology were, for decades, included into the Lecanora subg. Placodium sect. Placodium group. This section was proposed by Ryan and Nash (1993) for the *Lecanora* species characterised by an areolate-squamulose, lobate or subfoliose thallus, usually with a true cortex and loose medulla. Modern insights into the genus taxonomy afforded by molecular studies, however, revealed that thallus morphology in lecanoroid lichens does not reflect phylogenetic relationships. Moreover, the genus, *Lecanora* sensu lato, as well as subgenus, *Placodium*, turned out to be highly heterogeneous and polyphyletic (Poelt and Grube 1993; Arup and Grube 1998; Pérez-Ortega et al. 2010; Kondratyuk et al. 2014b; Leavitt et al. 2016). Still, the *Protoparmeliopsis* genus was not accepted as a separate genus in the family, Lecanoraceae, for a long time, based on the molecular data (Lumbsch and Huhndorf 2007, 2010). Recent studies have identified it as a well-supported, monophyletic clade nested within *Lecanora* s.l. and it has been subsequently posited to be accepted at the generic level (Kondratyuk et al. 2014b; Miadlikowska et al. 2014; Zhao et al. 2016).

During independent research, concentrated on the biodiversity of saxicolous lichens in Bolivia and Peru, as well as southern Poland, an interesting placodioid representative of Lecanoraceae has been found. Morphology and chemistry of the species suggested that it belongs to the *Protoparmeliopsis* genus. However, establishing its epithet turned out to be challenging. The scope of our study was to explain the systematic position of the lichen with application of integrated taxonomy tools. The survey revealed that the collection represents *P. garovaglii* and the status of the species is briefly discussed. As the epithet was never typified, a herbarium query was performed and, as a result, the species is neotypified herein.

## Material and methods

### Morphology and chemistry

This study is based on collections from the following herbaria: ASU, KRAM, L, MIN and WRSL, as well as the first author’s private material (hb. Szczepańska). The morphology and anatomy of the specimens were studied with a dissecting and light microscope according to routine techniques. For light microscopy, vertical, free-hand sections of apothecia were cut by a razor blade and mounted in water. Hymenium measurements were made in water and ascospores measurements in 10% potassium hydroxide – KOH (K). The structure and conglutination of paraphyses were also studied in K. The solubility of granules in epihymenium was tested with K and N (50% nitric acid). At least 10 measurements of the morphological variables were made for each sample and 20 spores from different specimens were assessed, as well as their minimum and maximum values being calculated.

Chemical examination included colour reactions and thin-layer chromatography (TLC). Spot test reactions of thalli, apothecial margins and discs were made with K, sodium hypochlorite [commercial laundry bleach] (C) and paraphenylenediamine [solution in 95% ethyl alcohol] (PD). The TLC analyses were undertaken in solvent system A, B’ and C using the standardised method of Culberson (1972) and following Orange et al. (2001).

Descriptions of the species are based on our own observations, measurements and TLC analyses made while examining the specimens cited in this paper. All specimens presented in the manuscript as in “Specimens examined” and included in the molecular analysis were studied; however, the morphological description of *Protoparmeliopsis garovaglii* is primarily based on the proposed neotype specimen. The terminology used in the descriptions of the species is based on Ryan et al. (2004).

### DNA extraction, amplification and sequencing

Genomic DNA was extracted from lichen thalli using the CTab method (Cubero and Crespo 2002). Dried tissues were frozen using liquid nitrogen and disrupted using Mixer Mill MM400 (Retsch; Haan, Germany). The isolated DNA was visualised on 1% TBE agarose gel. The fungal Internal Transcribed Spacer (ITS) rDNA region, which is a commonly used universal barcode marker in studies of non-lichenised and lichenised fungi, has been used in our study. ITS rDNA regions were amplified using primers ITS1F (Gardes and Bruns 1993) and ITS4 (White et al. 1990). The PCR reaction mix included (in the total volume of 20 µl): 1U Taq recombinant polymerase (Thermo-Fisher Scientific, USA), 10X Taq Buffer, 1 mM MgCl_2_, 0.5 µM of each primer, 0.4 mM dNTP and 1 µl DNA template. The PCR cycle was undertaken with a Veriti Thermal Cycler (Life Technologies; Carlsbad, CA, USA) with the following parameters: 8 min at 95 °C, followed by 32 cycles: 45 s at 95 °C, 45 s at 52 °C (annealing), 1 min at 72 °C, with a final extension step of 10 min at 72 °C. Prior to sequencing, PCR products were purified using GeneMATRIX PCR/DNA Clean Up Purification Kit (Eurx; Gdańsk, Poland). Sequencing, post-reaction purification and readings were undertaken by the sequencing service Genomed (Genomed S.A.; Warsaw, Poland), using an ABI 377XL Automated DNA Sequencer (Applied Biosystems; Carlsbad, CA, USA).

### Phylogenetic analysis

The obtained ITS rDNA sequences were assembled and manually edited using Geneious Pro, version 8.0. (Biomatters Ltd) and we also compared our fragments against the BLAST database in order to avoid potential contamination of other fungi (Altschul et al. 1990). We selected ITS sequences of *Protoparmeliopsis garovaglii*, *P. achariana*, *P. macrocyclos*, *P. muralis*, *P. peltata*, *P. zareii* and related genera (*Myriolecis*, *Protoparmelia* and *Rhizoplaca*), newly obtained in this study or downloaded from GenBank. Detailed information regarding sequences including GenBank accession numbers and specimen localities are found in Table 1. Subsequently, the final alignment was performed on the GUIDANCE 2 webserver (Sela et al. 2015) using the MAFFT algorithm (Katoh et al. 2005). The unreliable sites were removed (ca. 90% of sites remain in the alignment) in order to reduce errors caused by ambiguous sites (Penn et al. 2010). The nucleotide substitution models were separately searched for each subset of the partition of the ITS region (ITS1, 5.8S, ITS2) to find the best-fitting model using the corrected Akaike information criterion (AICc) as an optimality model criterion for a greedy algorithm search, as implemented in PartitionFinder version 1.0.1 (Lanfear et al. 2012).

**Table 1.** The species and specimens studied; newly generated sequences for this study are in bold.

Species	Isolate	Locality	Collector (-s)	Voucher specimens (herbarium)	GenBank no. (ITS)
* Myriolecis contractula *	AFTOL-ID 877	USA, Washington country	Brodo	Brodo 31501 (DUKE)	HQ650604
* Myriolecis dispersa *		USA, Illinois	Leavitt	Leavitt 12-002 (BRY-C)	KT453733
		Unitet Kingdom	Hill *s.n.*		KT453734
* Protoparmeliopsis achariana *				U155	AF070019
* Protoparmeliopsis garovaglii *		Austria			AF189718
	78	USA, Idaho	Leavitt	Leavitt 078 (BRY-C)	KU934540
	88	USA, Idaho	Leavitt	Leavitt 078 (BRY-C)	KU934541
	89	USA, Idaho	Leavitt	Leavitt 079 (BRY-C)	KT453728
	95	USA, Idaho	Leavitt	Leavitt 095 (BRY-C)	KU934542
	104	USA, Idaho	Leavitt	Leavitt 104 (BRY-C)	KU934544
	105	USA, Idaho	Leavitt	Leavitt 105 (BRY-C)	KU934545
	106	USA, Idaho	Leavitt	Leavitt 106 (BRY-C)	KU934546
	107	USA, Idaho	Leavitt	Leavitt 107 (BRY-C)	KU934547
	108	USA, Idaho	Leavitt	Leavitt 108 (BRY-C)	KU934548
	109	USA, Idaho	Leavitt	Leavitt 109 (BRY-C)	KU934549
	110	USA, Idaho	Leavitt	Leavitt 110 (BRY-C)	KU934543
	116	USA, Idaho	Leavitt	Leavitt 116 (BRY-C)	KU934550
	139	USA, Utah	Leavitt	Leavitt 139 (BRY-C)	KU934551
	140	USA, Utah	Leavitt	Leavitt 140 (BRY-C)	KU934535
	142	USA, Utah	Leavitt	Leavitt 142 (BRY-C)	KT453729
	142	USA, Utah	Leavitt	Leavitt 142 (BRY-C)	KU934536
	145	USA, Utah	Leavitt	Leavitt 145 (BRY-C)	KT453727
	199	USA, Utah	Leavitt	Leavitt 199 (BRY-C)	KU934537
*** Protoparmeliopsis garovaglii ***	**L21**	**Poland**	**Szczepańska**	**Szczepańska 1240 (WRSL)**	**MK084624**
	**L88**	**Bolivia**	**Flakus**	**Flakus 17529 (KRAM)**	**MK084625**
	**L89**	**Bolivia**	**Flakus**	**Flakus 21175 (KRAM)**	**MK084626**
	**L90**	**Bolivia**	**Flakus**	**Flakus 21118 (KRAM)**	**MK084627**
	**L91**	**Peru**	**Flakus**	**Flakus 9540 (KRAM)**	**MK084629**
	**L92**	**Peru**	**Flakus**	**Flakus 9603 (KRAM)**	**MK084628**
* Protoparmeliopsis macrocyclos *		Sweden		U273	AF159933
* Protoparmeliopsis muralis *				M122	AF070015
	DNA 9890	Germany, Saxony	Scholz	Scholz 0275697 (M)	KT818623
	SK 765	Romania	J.-S. Hur	J.-S. Hur (RO11-130) KOLRI	KP059048
		Russia	Vondrak	Vondrak 106a (PRA)	KU934559
		Russia	Vondrak	Vondrak 106b (PRA)	KU934560
		Russia	Vondrak	Vondrak 9405 (PRA)	KU934556
		Russia	Vondrak	Vondrak 9417 (PRA)	KU934557
		Russia	Vondrak	Vondrak 9417 (PRA)	KT453724
	77	USA, Utah	Leavitt	Leavitt 077 (BRY-C)	KU934552
	141	USA, Utah	Leavitt	Leavitt 141 (BRY-C)	KT453725
	143	USA, Utah	Leavitt	Leavitt 143 (BRY-C)	KU934554
* Protoparmeliopsis peltata *		Iran	Sohrabi	MS014622	KT453723
		Iran	Sohrabi	MS014620 (personal herbarium)	KU934739
		Iran	Sohrabi	MS014621pelt (personal herbarium)	KU934721
		Iran	Sohrabi	MS014623 (personal herbarium)	KU934722
		Iran	Sohrabi	MS014624pelt (personal herbarium)	KU934723
		Iran	Sohrabi	MS014630 (personal herbarium)	KU934731
		Iran	Sohrabi	MS014637 (personal herbarium)	KU934732
		Iran	Sohrabi	MS014638 (personal herbarium)	KU934733
		Kazakhstan		Kaz 12921c	KU934745
		Kazakhstan		Kaz 13085pelt	KU934746
		Kazakhstan		Kaz 12943	KU934747
		Kazakhstan		Kaz 12948	KU934748
		Kazakhstan		Kaz 13082	KU934749
		Kyrgyzstan	?Lommi, Sampsa	H920340	KU934720
		Kyrgyzstan		H9203329	KU934719
		Kyrgyzstan		H9203118	KU934735
		Kyrgyzstan		H9203304	KU934736
		Kyrgyzstan		H9203334	KU934737
		Kyrgyzstan		H9203194	KU934738
		Russia	Vondrak	Vondrak 9987 (PRA)	KU934725
		Russia	Vondrak	Vondrak 9997 (PRA)	KU934726
		Russia	Vondrak	Vondrak 10016 (PRA)	KU934727
		Russia	Vondrak	Vondrak 10022 (PRA)	KU934728
		Russia	Vondrak	Vondrak 10041 (PRA)	KU934729
		Russia	Vondrak	Vondrak 10130 (PRA)	KU934730
		Russia	Vondrak	Vondrak 9423 (PRA)	KU934740
		Russia	Vondrak	Vondrak V127 (PRA)	KU934751
		Russia	AsLap	951	KU934742
		Russia	AitLap	876	KU934744
		Russia	Sar	937	KU934743
		Turkey	Vondrak	Vondrak 9783 (PRA)	KU934724
		USA	Leavitt	Leavitt 601 (BRY-C)	KU934734
		USA	Leavitt	Leavitt 663 (BRY-C)	KU934741
	U198	USA, Arizona		cf. ASU	AF159925
		USA, Utah			KT453722
* Protoparmeliopsis zareii *	480	Iran	B. Zarei-Darki	Zarei-Darki 1111 (SK)	KP059049
	480	Iran	B. Zarei-Darki	Zarei-Darki 1111 (SK)	KP059049

The phylogenetic construction was generated using the Maximum Likelihood (ML) bootstrap tree with simultaneous heuristic search, as implemented in RaxmlGUI version 0.9 beta 2 (Stamatakis 2006; Silvestro and Michalak 2012) under the GTRGAMMA substitution model and 200 bootstrap re-samples. Bayesian Inference was carried out with Markov Chain Monte Carlo (MCMC) implemented in MrBayes v3.2.3 (Ronquist et al. 2011). MrBayes was set to three independent parallel runs, each with four incrementally heated chains started, the run length was settled to 40M generations and, to infer convergence, the average standard deviation of the split frequencies was printed every 1000^th^ generation, discarding the first 50% of the trees sampled as a burn-in fraction. The analyses were stopped after 1M generations when the standard deviation had dropped below 0.01. The resulting phylogenetic trees were visualised in Figtree software (Rambaut 2014).

## Results

### Phylogeny

**Figure 1.**
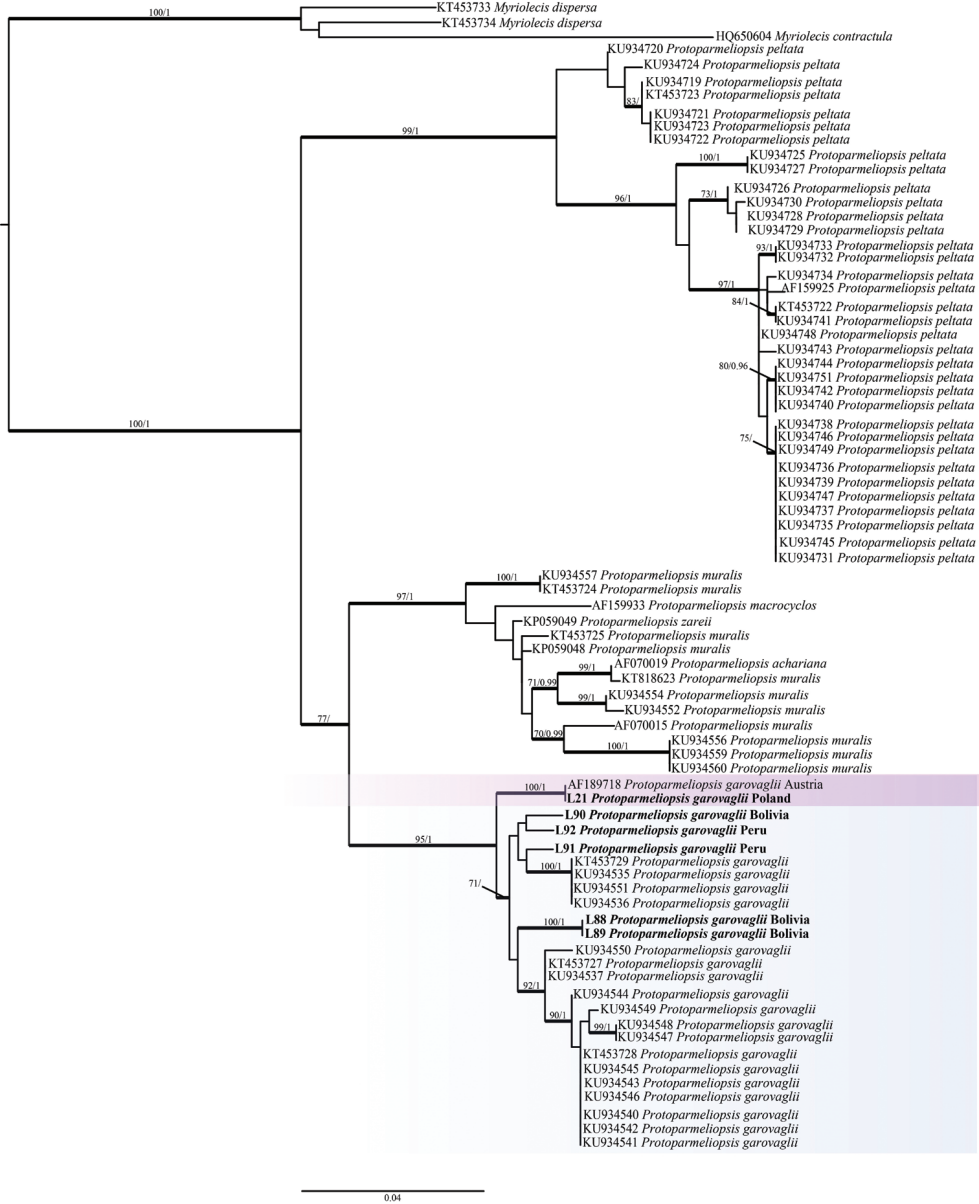
Bayesian Inference of the phylogenetic relationship within *Protoparmeliopsis* species, based on ITS rDNA sequences. High bootstrap support values are shown above thickened branches and bold numbers representing clades (ML – BP ≥ 70%, Bayesian analysis – PP ≥ 0.9). Highlighted squares represent *P. garovaglii* populations in Europe, South and North America. Parmeliaceae species were selected as the outgroup.

A total of 77 sequences were analysed in this study. The final alignment matrix contained eight OTUs and 545 unambiguously aligned nucleotides positions. The phylogeny shows highly supported clades [bootstrap support (BS) = 75%, posterior probability (PP) = 1] inferred from a single locus phylogeny, clearly delimiting the Lecanoraceae as separate from *Myriolecis* (outgroup) (Fig. 1). *P. garovaglii* forms a monophyletic clade highly supported (BS = 95%, PP = 1) within *Protoparmeliopsis*. The newly generated sequence from Poland is placed in a monophyletic clade [BS = 100%, PP = 1] together with the Austrian sequence. South American (Bolivian and Peru; for the first time molecularly confirmed in this study) and USA populations are placed in different clades but lack statistical support.

### Taxonomy

#### Protoparmeliopsis garovaglii

Taxon classificationFungiLecanoralesLecanoraceae

(Körb.) Arup, Zhao Xin & Lumbsch; Fungal Diversity 78: 301 (2016) [2015].

07f7efe6-55ad-5cb4-a9e2-d7a282b3358d

Mycobank: 387928

Figs 2a–b

##### Basionym.

*Placodium garovaglii* Körb., Parerga Lichenol. (Breslau) 1:54 (1859) ≡ *Squamaria garovaglii* (Körb.) Anzi, Cat. Lich. Sondr. 46 (1860) ≡ *Lecanora garovaglii* (Körb.) Zahlbr., Ann. Naturhist. Hofmus. 15:208 (1900) ≡ *Placolecanora garovaglii* (Körb.) Räsänen, Hedwigia 81:230 (1944).

##### Type.

Hungary. Szent-György-hegy Mt, ‘Ad saxa basaltica montis “Szentgyörgyhegy” prope pagum Kisapáti, comit. Zala. Altit. ca. 400 m. s. m. Mens. Jun. 1920, G.Timkó’ [*Flora Hungarici exsiccata* 617, as *Lecanora garovaglii*] (neotype: WRSL-5777, designated here).

##### Description.

**Figure 2.**
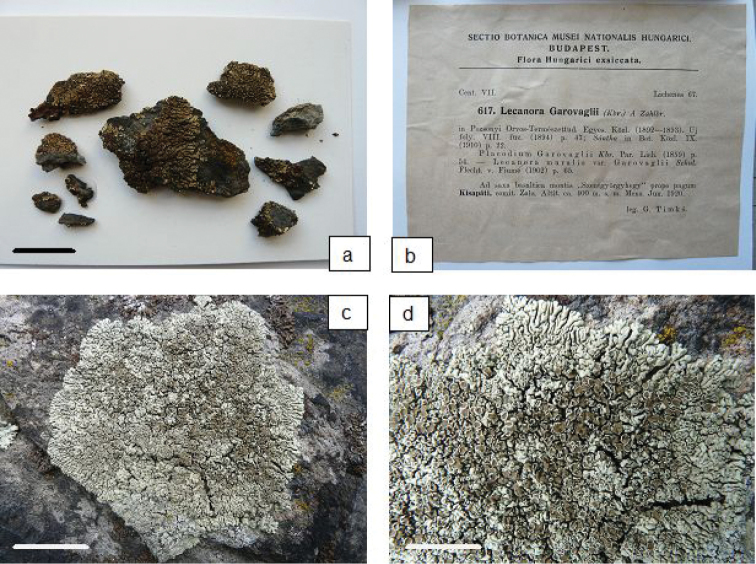
**a, b** Neotype for *Protoparmeliopsis garovaglii* designated in WRSL herbarium **c, d** Thallus of *Protoparmeliopsis garovaglii* in natural habitat. Scales: 2 cm (**a**); 4 cm (**c**); 2 cm (**d**).

Thallus lichenised, placodioid, thick, usually distinctly circular, up to 12 cm diam., not very closely attached to the substrate, prothallus not present. Marginal lobes elongated, distinctly convex, swollen, sinuous, smooth 0.4–1.8 mm wide and 3–10 mm long, broadened and rounded at the ends (Figs 2c–d). Thallus centre more or less areolate. Areoles convex, irregular, overlapping, 0.25–1.0 mm diam. Upper surface mat, pale yellowish-green to greyish-green, tending to be darker in the central part of the thallus, sometimes shining and darker also at the edges of the marginal lobes. Lower surface pale brown. Medulla white, in older lobes distinctly hollow in the middle part. Apothecia sessile to constricted at base, dispersed to clustered towards thallus centre, 0.5–2.0 mm diam., circular, older angular, proper margin persistent, paler or concolorous with thallus, matte, slightly radially cracked, flexuose in older and disappearing in mature apothecia. Disc pale brown to yellowish-brown, becoming darker in the centre of thallus, epruinose, flat. Hymenium colourless, 50–60 μm high, hypothecium colourless, epihymenium orange-brown with small granules soluble in K and insoluble in N. Asci clavate, eight-spored. Paraphyses simple or weakly branched with swollen apices. Ascospores hyaline, simple, ellipsoid to oblong-elipsoid, 10–12 × 6–7 μm. Pycnidia not seen.

##### Chemistry.

thallus K+ pale yellow, C–, KC+ yellow, P–; medulla K+ pale yellow, C–, KC+ yellow, P–. Secondary metabolites detected by TLC: ± isousnic, +usnic and ±placodiolic acids (cortex); +zeorin and ± unidentified terpenoides (medulla).

##### Distribution.

the species is widely distributed in the world. It occurs in Europe, Asia, Africa (Morocco; Egea 1996), North America (Canada; Freebury 2014 and USA; Ryan et al. 2004) and South America (Feuerer et al. 1998; Feuerer and Sipman 2005). In Asia, it has been noted in Afghanistan (Poelt and Wirth 1968), India (Upreti and Chatterjee 1998; Singh and Sinha 2010), Iran (Sohrabi et al. 2010), Mongolia (Schubert and Klement 1971), Pakistan (Poelt 1961), Russia (Vondráková and Vondrák 2015), Tajikistan (Kudratov and Mayrhofer 2002) and Turkey (Halici and Candan 2007). In Europe, its records are derived so far from Armenia (Gasparyan et al. 2016), Austria (Hafellner and Türk 2001), the Czech Republic (Vězda and Liška 1999), Germany (Wirth 1995), Greece (Grube et al. 2001), Italy (Nimis 2016), Netherlands (Aptroot 2011), Portugal and Spain (Llimona and Hladun 2001), as well as Ukraine (Kondratyuk et al. 1996). Here, the species is reported for the first time from Poland.

##### Ecology.

*Protoparmeliopsis garovaglii* is widespread, occurring mostly in dry and warm Mediterranean to mountain areas, foothills and submontane sites (Ryan et al. 2004). It prefers slightly calcareous or basic silicate rocks (limestone, basalt, rhyolite, schist, pumice, volcanic ash, sandstone) and usually occupies sunny habitats, especially steep surfaces (Wirth 1995; Ryan et al. 2004). However, it is noteworthy that, on its northernmost locality in the Netherlands, the species was recorded on a tombstone (Aptroot 2011). In Poland, it was found in mountain areas with outcrops of basalt rocks in the form of a volcanic chimney, surrounded by granite casing. It occupied a lit, warm and dry place on the horizontal surface of the basalt rock with a southern exposure and was accompanied by other lichens such as *Acarospora fuscata*, *Candelariella coralliza*, *Protoparmeliopsis muralis*, *Lecanora rupicola* and *Rhizocarpon geographicum*. During the present study in Bolivia and Peru, *P. garovaglii* was recorded in high Andean open-vegetative regions and in open semi-desert montane areas.

##### Exiccates examined.

Pišut, *Lichenes Slovakiae exsiccati* 36, as *Lecanora garovaglii* (KRAM); Suza, *Lichenes Bohemoslovakiae exiccati* 233, as *Lecanora garovaglii* (KRAM); Weber, *Lichenes exsiccati* 118, as *Lecanora garovaglii* (KRAM).

##### Specimens examined.

Poland. Przedgórze Sudeckie foreland: Wzgórza Strzegomskie hills, Góra Świętego Jerzego Mt, 50°58'25"N, 16°20'10"E, on basalt rocks, 354 m alt., 4 Oct. 2013, K.Szczepańska 1240 (WRSL). Bolivia. Dept. La Paz, Prov. Bautista Saavedra: Anmin Apolobamba, near Taypi Cañuma village, 15°03'20"S, 69°09'07"W, 4506 m alt., 5 July 2010, A.Flakus 17529 & P.Rodriguez-Flakus (KRAM, LPB); on the road from Apolo to Charazani villages (162 km), la Cruz Charazani-Pelechuco, 15°15'00"S, 69°02'51"W, 4545 m alt., 19 May 2011, A.Flakus 21118, 21175, 21176 & O.Plata (KRAM, LPB). Peru. Cañon del Colca, Dept. Arequipa, Prov. Caylloma: near Cabanaconde village, 15°37'56"S, 71°57'49"W, 3462 m alt., 4 July 2006, A.Flakus 9540 (KRAM); *ibid.*15°38'18"S, 71°57'43"W, 3480 m alt., 5 July 2006, A.Flakus 9603 (KRAM).

##### Additional specimens examined.

Austria. Lower Austria: sunny slate rocks near Krems on the Danube River, 250 m alt., 3 Jan. 1897, Baumgarten (L). USA. Arizona. *Coconino Co*.: Grand Canyon National Forest, top of Hermit Trail, pinyon-juniper woodland, on limestone, 1950 m alt., 11 July 1994, T.H.Nash III 35474 (ASU); *ibid.*, South Kaibab Trail, on sandstone, 1950 m alt., 29 June 1991, M.Boykin 2053 (ASU); *Greenlee Co*.: Apache National Forest, Juan Miller Canyon camp-ground, along the Blue River, ponderosa pine forest with riparian sp., on acid rock, 1740 m alt., 6 June 1998, T.H.Nash III 41809 (ASU); *Maricopa Co*.: Crater Range, along AZ 85, 42 km S of Gila Bend Sonoran Desert, on granite, 425 m alt., 27 Feb. 1998, T.H.Nash III 40608 (ASU); *Santa Cruz Co*.: Coronado National Forest, hillsides to S of Pena Blanca Lake (ca. 15 km WNW of Nogales) and just S of Ruby-Nogales Rd., oak woodland steep slope with rhyolite, on rhyolite, 1200 m alt., 2 June 1998, T.H.Nash III 41656 (ASU). Idaho. *Twin Falls Co*.: E side of U.S. Hwy 30, 6.8 km S of Bills, on basalt, 915 m alt., 11 Sept. 1998, B.D.Ryan 32953 (ASU). Nevada. *Churchill* Co.: US Hwy 50, N end of Desatoga Mountains, 84 m E of Fallon, 1830 m alt., July 1984, B.D.Ryan 11554 (MIN). North Dakota. *Billings Co*.: Theodore Rooselvelt Nat. Park, S. Unit One mile S of Paddock Creek along park road, on ridge E of road on scoria rock, 2500 ft. alt., 25 July 1982, C.Wetmore 45128 (MIN). Montana. *Park Co*.: Yellowstone National Park, Grazing enclosure 1 mile W of Gardiner at northern edge of park, open grassland on knoll with sagebrush and rock outcrop, 5300 ft. alt., 21 July 1998, C.Wetmore 80972 (MIN).

## Discussion

*Protoparmeliopsis garovaglii* was traditionally characterised by its typically elongate and strongly inflated-plicate lobes of the thalli. For most details, the species was studied by Ryan and Nash (1993), who treated it as a single frequent widespread and extremely variable taxon – *Lecanora garovaglii* s.l., including *L. cascadensis* H. Magn., *L. nevadensis* H. Magn. and *L. peruviana* (Müll. Arg.) Zahlbr. By examining hundreds of specimens, the authors were deeply involved in discussions about the species’ variety concerning colour of apothecial discs and associated epihymenial features. They finally concluded that the set of mentioned phenotypic traits is often not clearly expressed and does not exhibit clear correlations with other characters, such as secondary chemistry. Moreover, both disc colour and cortical chemistry correlate with habitat and distribution, respectively, rather than directly with each other. According to us, this serves as evidence of possible phenotypic plasticity, not taxon speciation. The cortical chemistry variation throughout the geographical range of *L. garovaglii* with three cortical substances (isousnic, usnic and placodiolic acids) in different combinations is a separate, interesting problem, discussed in the paper by Ryan and Nash (1993) and ending with the statement that the name cannot be unambiguously assigned to any of the known chemotypes as it is not typified. In this situation, the authors referred to the only specimen under the name, *Placodium garovaglii*, available at that time in the Körber “Typenherbar” in L, originating from “Vel Furva” (Valfurva city, Italy) and containing isousnic and usnic acids in the cortex. However, Körber’s collection is kept in the Leiden Herbarium as two different parts. Specimens from the first (Hauptsammlung) are labelled as “Koerber Stammherbar” and those from the second (Typensammlung) as “Koerber Typenherbar” (Liška 2013). It is not clear if Ryan and Nash (1993) searched for original material in both collections or only in the “Typenherbar”.

During our study, we tried to trace the original collection of the species. Type citation in the protologue is: ‘An basaltigem Gestein “in monte supra Varzi” von Garovaglio gesammelt (Herb. Heufl.)’ [Italy, Prov. Pavia, Region of Lombardy, the mountain above Varzi city, on basalt rock, leg. Garovaglio] (Körber, 1859–1865). Heufler’s herbarium was sold after his death and currently the final destination of the samples is unknown. We started our enquiries at IBF where Haufler deposited much of his herbarium material during his lifetime. This did not bring any resolution as our double request did not elicit a response. We also requested the specimens of *P. garovaglii* from L herbarium. Subsequent to the request, we received the historical collection of *P. garovaglii* from the locality: Lower Austria, sunny slate rocks near Krems on the Danube River, alt. 250 m, 3 Jan. 1897, leg. Baumgarten. Obviously, the species cannot be lectotypified, as there is only one locality cited in the protologue and the original collection of the species from *locus classicus* could not be located at any herbaria and may have been lost. For name typification, we considered the collection available at L, however, its lowland origin and cortical chemistry (usnic and placodiolic acids) indicate that it would not be the best choice. We have also made a request at WRSL herbarium knowing that some small part of Körber’s collection is also located there. However, none of Körber’s specimens representing *P. garovaglii* was available. The most appropriate material for the neotype of the historical collections seen by us is apparently the exsiccate from WRSL, collected in the mountain area of Hungary and it was designated there. This specimen is well preserved, was collected from the basalt rock, has typical morphology suitable to the description given in the protologue and the following cortical chemistry: isousnic, usnic and placodiolic acids (the most frequent chemotype in Europe, according to Ryan and Nash (1993)).

The species most closely related and likely to be confused with *P. garovaglii* is *P. muralis*. In contrast to *P. garovaglii*, the thallus of *P. muralis* is smaller and much more strongly attached to the substrate. Furthermore, thallus lobes of the latter species are distinctly shorter, flattened and thinner and not swollen or sinuous-plicate as they are in the case of *P. garovaglii*. Both species can also be distinguished by their chemistry. *Protoparmeliopsis muralis* contains usnic acid and zeorin but also atranorin, leucotylin, murolic and psoromic acids; the latter are not produced by *P. garovaglii* (Wirth 1995; Ryan et al. 2004; Edwards et al. 2009). To some extent, *P. garovaglii* may also be mistaken with *Rhizoplaca subdiscrepans* (Nyl.) R. Sant., especially as both species have similar colour of the upper surface of the thallus and prefer similar, warm and dry habitats (Wirth 1995; Hafellner and Türk 2001). However, in contrast to *P. garovaglii*, the thallus of *R. subdiscrepans* is usually verrucose-squamulose, polyphyllous, without distinct lobes at the margin and pruinose apothecial discs (Ryan 2001). Both species also have similar cortical chemistry with isousnic, usnic and placodiolic acids in the upper cortex, but *P. garovaglii* additionally contains zeorin in the medulla.

*Protoparmeliopsis garovaglii* was included in previous phylogenetic frameworks focused on European, North American and Asian populations (Arup and Grube 1998, 2000; Leavitt et al. 2016; Kondratyuk et al. 2014a, b). In this study, we included new sequences from South America and they are placed in a single, highly supported, species-level lineage (BS = 100%, PP = 1). There is a geographical differentiation tendency based on our molecular output. The Polish specimen is placed in a monophyletic clade with a highly supported group (BS = 100%, PP = 1) together with the Austrian sequence. Bolivian, Peruvian and North American populations are placed in different clades but, in most cases, the internal node lacks statistical support. This tendency may follow a population geographical disjunction of different organisms, including lichens, in which the morphological and chemical characters are highly variable in a single species, making a real challenge for species delimitation and, in most cases, these species are treated as a ‘complex’. In the case of lichenised fungi, some previous extensive studies on molecular population or/and phylogeography analyses on species recognition boundaries, such as *Usnea perpusilla* (Wirtz et al. 2008), *Leptogium furfuraceum* (Otálora et al. 2010) *Xanthoparmelia pulla* (Amo de Paz et al. 2012), were performed.

In our study, we analysed differences in morphology, anatomy and chemistry of specimens representing different clades. European material is characterised by a pale green colour of the thallus with elongated, distinctly convex and swollen marginal lobes, which is not very closely attached to the substrate. The apothecial discs are epruinose, bright to dark brown in colour. Within material originating from Bolivia and Peru, we found very similar morphology of the apothecia and thallus, however the thallus colour of Bolivian specimens is more pale yellow than green. In North American, the thallus in many cases is smaller and more closely attached to the substrate, with flat, shorter and narrower marginal lobes (0.3–1.2 mm wide and 2–6 mm long) and is additionally pruinose at the ends. The colour of the discs is usually brown but also yellow-green or yellow-orange, when the upper surface of the thallus has more orange tint. No significant differences were found in the colour or height of the hymenium and epihymenium, nor the paraphyses or shape and size of spores in the specimens representing different clades. Furthermore, we have not found any correlation between secondary chemistry of the thallus and species distribution. Both specimens from Europe, South and North America (Bolivia, Peru and USA) contain zeorin and usnic acids as solid components, when isousnic and placodiolic acids, as well as unidentified terpenoides may be present or absent; however, no sample from South America contained isousnic acid.

Based on these observations, we may confirm great phenotypic variation of specimens representing *P. garovaglii* s.l., also observed by Ryan and Nash (1993). However, we cannot unambiguously correlate perceivable morphotypes with appropriate clades. In particular, morphological differentiation may also greatly reflect responses of individuals to diversity of habitat conditions. Moreover, any far-reaching conclusions must be based on a larger sampling size and should be statistically supported.

We do not claim to assign any taxonomic resolutions concerning *P. garovaglii* s.l. until further molecular population studies provide evidence for species delimitation within the species-complex. The intention of the current study was to genetically support the identification of *P. garovaglii* in collections from areas of research interest to the authors. As a result, molecular evidence of the species occurrences in Poland and South America (Bolivia and Peru) was supplied. Typification of the epithet *P. garovaglii*, via this work, should be useful for further circumscription of related taxa.

## Supplementary Material

XML Treatment for Protoparmeliopsis garovaglii
